# Genomic Characterization of a Set of Iberian Peninsula Bovine Local Breeds at Risk of Extinction: Morenas Gallegas

**DOI:** 10.3390/ani10111956

**Published:** 2020-10-23

**Authors:** María Asunción García-Atance, Carlos Carleos, Susana Dunner, Paulina G. Eusebi, Castor José Rivero, José Ramón Justo, Miguel Fernández, Javier Cañón, Óscar Cortés

**Affiliations:** 1 Universidad Alfonxo X El Sabio, Avda. Universidad, 1., 28691 Villanueva de la Cañada, Madrid, Spain; agatance@yahoo.es; 2 Dpto. Estadística e Investigación Operativa, Universidad de Oviedo, 33007 Oviedo, Spain; carleos@uniovi.es; 3 Departamento de Producción Animal, Facultad de Veterinaria, Universidad Complutense de Madrid, 28040 Madrid, Spain; dunner@vet.ucm.es (S.D.); jcanon@vet.ucm.es (J.C.); 4 VELOGEN.SL. Servicio de Genética, Facultad de Veterinaria, Universidad Complutense, Avenida Puerta de Hierro, s/n, 28040 Madrid, Spain; pau_g_e@hotmail.com; 5 Centro de Recursos Zooxenéticos de Galicia, 32152 Fontefiz, Ourense, Spain; boaga@boaga.es; 6 Federación de Razas Autóctonas de Galicia-BOAGA, 32152 Fontefiz, Ourense, Spain; frag@boaga.es; 7 Centro Tecnológico de la Carne de Galicia, 32900 San Cibrao das Viñas, Ourense, Spain; mfr_barra@yahoo.es

**Keywords:** genomic diversity, Morenas Gallegas bovine breeds, Singel Nucleotide Polymorphisms (SNPs), biodiversity conservation, Caldelá, Cachena, Frieiresa, Limiá, VIanesa

## Abstract

**Simple Summary:**

Genetic characterization of breeds at risk of extinction is crucial in order to develop efficient conservation plans. In this study, for the first time to our knowledge, a set of bovine breeds summarized as Morenas Gallegas (Cachena, Caldelá, Frieiresa, Limiá, and Vianesa), located in the Northwest of Spain, were studied by using genomics tools. Our aim was to analyze their genetic diversity and their genetic relationships with a set of local and cosmopolitan European bovine breeds. The results evidence the specific genetic signature of the Morenas Gallegas bovine breeds. Cachena was the only breed located in the same cluster as the Spanish bovine breeds analyzed, while Caldelá, Frieiresa, Limiá, and Vianesa were clearly separated. The genetic diversity of the set of Morenas Gallegas breeds is intermediate or high, but Frieiresa breed showed the higher value of inbreeding coefficient and requires special attention in the next years. Vianesa shows two different lineages, with an evident Frieiresa component and without it. Our results are the proof of the valuable genetic reservoir that Morenas Gallegas bovine breeds is due to their uniqueness in comparison with other Spanish bovine breeds.

**Abstract:**

A set of five local bovine breeds in danger of extinction named Cachena, Caldelá, Limiá, Frieiresa, and Vianesa and included in the group of Morenas Gallegas are located in the Autonomous Community of Galicia at the Northwest of Spain. Local authorities launched a conservation plan at the end of the 21th century in order to preserve this important genetic reservoir. However, Morenas Gallegas bovine breeds never have been analyzed with genomic tools and this information may be crucial to develop conservation plans. The aim of the study was to analyze their genetic diversity and genetic relationships with a set of local and cosmopolitan European bovine breeds using single nucleotide polymorphisms. Our results show own genetic signatures for the Morenas Gallegas breeds which form a separate cluster when compared to the Spanish breeds analyzed, with the exception of the Cachena breed. The genetic diversity levels of the Morenas Gallegas were intermediate or high, and low inbreeding coefficients can be found except for the Frieiresa breed (11%). Vianesa breed evidenced two lineages depending on the Frieiresa component influence. The Morenas Gallegas bovine breeds group represent an important Spanish bovine genetic reservoir and despite their classification within a single generic group, the five breeds show their own genetic uniqueness.

## 1. Introduction

Livestock populations have experienced an alarming rate of genetic erosion in the last three decades. Approximately, 200 breeds have disappeared and up to 30% are endangered [1]. This trend had a higher impact in local breeds that have been replaced by high production ones all over the world [2]. Rare breeds are distributed and well adapted to a wide variety of local environmental conditions, however, the continued loss of genetic diversity in last decades may jeopardize the ability of the bovine species to adapt to changing environmental conditions or to fulfil specific requirements that could be necessary in the future. It is remarkable that over the last recent years a great number of local breeds have become extinct [3]. At present, 32 of the 46 Spanish bovine breeds officially recognized are classified as endangered (www.mapama.es), and national actions have been established in order to preserve that important genetic resource recognizing the special value of endangered local breeds (Royal Decree 2008).

**Figure 1:**
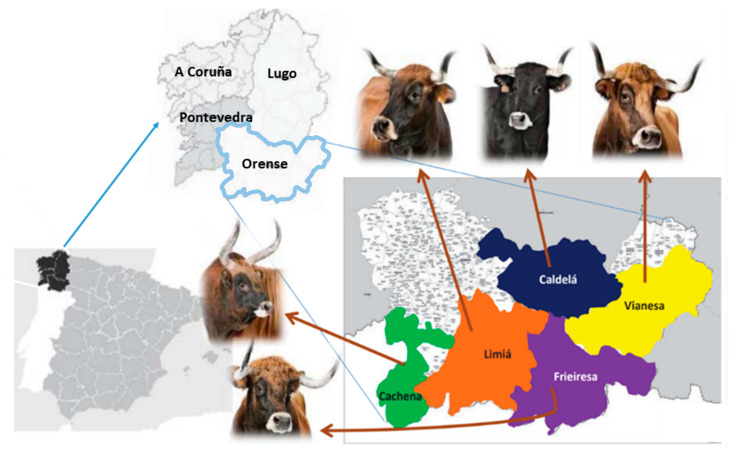
Geographic location of Morenas Gallegas bovine breeds: Cachena, Caldelá, Frieiresa, Limiá, and Vianesa.

The region of Galicia, in the Northwest of the Iberian Peninsula, hosts a set of local cattle breeds classified as endangered called Morenas Gallegas (Figure 1). This geographic region has a long history of livestock farming. In the 17th century, small family farms in Galicia held estimations of almost one million of bovine individuals and until the year 1891 there were no foreign breeds in Galicia. Those animals served for multiple purposes, as milk and meat production but also for work or cultural activities [4]. The first reference to this set of breeds called Morenas del Noroeste (Norwest Morenas) was in the late 19th century [5]. Since 1992, the name changed to Morenas Gallegas [6], which includes five bovine breeds: Cachena, Caldelá, Limiá, Vianesa, and Frieiresa [7]. In addition to national actions, the local government (Xunta de Galicia) launched in 1990 a conservation program for these breeds [8]. The combination of ex situ (genebank activities such as semen collection) and in situ (registration of animals belonging to these breeds in their original location to recover animal and farm census) actions prompted an increased tendency of their effective population size (Ne) since 1995 [9]. However, the demographic characteristics of these breeds are still critical as revealed by the fact that the breed with higher census was Cachena (4067 individuals—3881 females and 726 males in 2019) while Frieiresa is under 1000 individuals (750 females, 133 males) (Table 1). As these local breeds are well adapted to the traditional regional farming system and contribute to the preservation of the rural environment, landscape and ecosystem services, their maintenance is crucial. A brief description of the five Morenas Gallegas bovine breeds can be found in annex 1.

To date the Morenas Gallegas breeds have not been included in any DNA study to elucidate the genetic structure and relationships between them and other European cattle breeds. Although, being located in a small geographic area distribution their belonging to the same racial group is unknown. Only a few studies with biochemical markers were carried out in the last decade of the 20th century in which this set of breeds and their relationships were analyzed with other Spanish cattle breeds [10,11]. The assessment of the genetic diversity of local breeds is an important task for conservation and management of local biodiversity to avoid the loss of an important genetic resource. Thus, the objective of the present study is to analyze the genetic diversity of the breeds called Morenas Gallegas using DNA markers and their genetic relationships with a comprehensive group of European bovine breeds. The results of the analysis will be an important tool to develop conservation programs in order to reserve this important genetic resource and to our knowledge has never been developed.

## 2. Materials and Methods

A total of 106 samples belonging to the five bovine breeds called Morenas Gallegas were analyzed in the study (Table 1). No ethical approval was required, in compliance with the European Directive 2010/63/UE because blood samples were taken during obligatory routine animal sanitary controls by an authorized veterinarian. Samples were genotyped using the Bovine SNP50K BeadChip V2 (Illumina, San Diego, CA, USA) and Affymetrix Axiom Bovine Genotyping v3. Common SNPs from both platforms were merged using Applied Biosystems™ Axiom™ Long Format Export (AxLE) Tool (Foster City, CA, USA), and resulting in 27,859 common autosomal genotyped SNPs that were used in the analysis. SNPs data were pruned using PLINK [12] (-maf 0.05, -geno 0.01, -mind 0.1).

Genetic diversity parameters, observed and expected heterozygosity, methods of moments inbreeding coefficient, and Principal Component Analysis (PCA) were estimated using PLINK [12]. Trends in historical effective population size (Ne) from linkage disequilibrium (LD) were estimated using SNeP V1.1 software [13].

Runs of Homozygosity (ROH) were detected as described in Cortes et al. 2019 [14] using PLINK [12] and three ROH of different lengths were defined >4, >8, and >16 Mb. For each animal, inbreeding based on ROH (F_ROH_) was calculated as the proportion of genome in ROH over the overall length of the genome covered by the involved SNPs (2,541,174 kb).

Individual ancestry proportions given a k number of ancestral populations were estimated using a maximum likelihood clustering approach implemented in ADMIXTURE [15]. For each ancestral K value five independents runs were performed for k = 2 to k = 5.

Pairwise allele sharing distance (ASD) among Morenas Gallegas individuals were estimated using ASD program (https://github.com/szpiech/asd). Reynolds’ genetic distances among individuals were estimated using ARLEQUIN software [16] and plotted in a Neighbor-net using the SPLITTREE software [17].

The software TREEMIX [18] was used to infer the presence of patterns of population splitting and introgression events. First, a maximum likelihood tree was constructed without migration events that were introduced subsequently, and admixture events were then introduced in the model (from 1 to 5) using Italian Brown Swiss as outgroup. The most predictive number of migration edges was selected using the optM function in the R package OptM [19].

In order to elucidate the genetic relationships among the Morenas Gallegas breeds and other local bovine breeds located in France, Italy, and the Iberian Peninsula, a total of 38 breeds were jointly analyzed. Table S1 describes the bovine breeds, number of samples, geographic location, and the source of the data used. Except for the Iberian Peninsula breeds Retinta, Avileña, Pasiega, Tudanca, and Asturiana de los Valles the remaining data were taken from public repositories. PCA, ADMIXTURE, TREEMIX, and Neighbor-net graph were constructed following the same rules as those described before.

## 3. Results

### 3.1. Genomic Diversity

Genomic diversity parameters and demographic information are shown in Table 1. The H_o_ was similar for the five breeds analyzed ranging from 0.32 (Frieiresa) to 0.36 (Caldelá), while the H_e_ was the same for all the breeds. Frieiresa showed the highest F_HOM_ average (0.11), ranging from 0.02 to 0.06 for the remaining breeds. As expected, Ne decreased as the number of generations increase. Frieiresa breed showed the lower historical Ne values until 27 generation ago while Cachena evidenced the maximum historical Ne values (Supplementary Figure S1). Assuming as contemporary effective size estimates relating to the 13 generations, Limiá breed shows the minimum value Ne (57), while Cachena the maximum value (78) (Table 1).

Individual genomic inbreeding was evaluated using ROH data. The distribution of the three genomic measures estimated is shown in Supplementary Figure S2. F_ROH_ decreased as the minimum ROH length increased. Frieiresa breed shows the highest F_ROH_ average for the three genomic measures while Cachena the lowest ones (Figure S2). Additionally, F_HOM_ and F_ROH_ 4Mb and F_ROH_ 8Mb were highly correlated, 0.99 and 0.89, respectively (Table S2).

In addition, the Galician Local Breeds Federation (BOAGA) provided us the effective population size adjusted to the genealogical information and the inbreeding coefficient of those animals born since 2010. Based on genealogical information Frieiresa, Limiá, and Vianesa evidenced similar Ne (22, 34, and 32, respectively) while Cachena and Caldelá’s are higher (65 and 74, respectively). Furthermore, inbreeding coefficients based on genealogical information for those animals born since 2010 ranged from 2.3% (Cachena) to 3.6% (Limiá and Vianesa) (Table 1).

### 3.2. Genetic Relationships

#### 3.2.1. Morenas Gallegas

**Figure 2:**
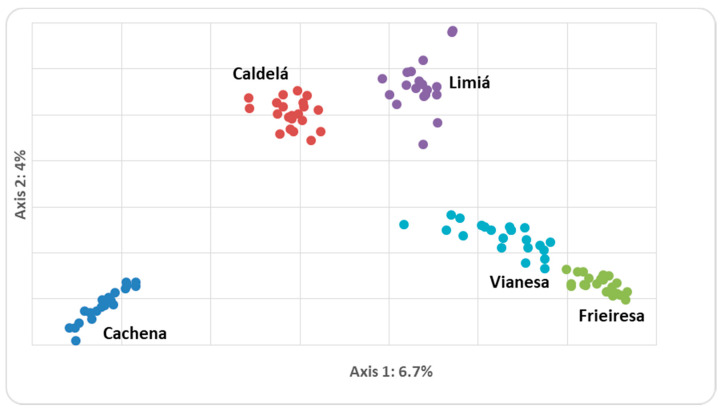
Spatial representation of the Morenas Gallegas bovine breeds analyzed from the first two axes obtained in the factorial analyses of correspondence.

PCA analysis revealed the genetic identity of the five bovine breeds (Figure 2). The first two axes cumulatively explained 11% of the total variance. Cachena, Caldelá, and Limiá formed three clearly separated clusters, while Frieiresa and Vianesa do not show overlapping positions but are very close to each other.

**Figure 3:**
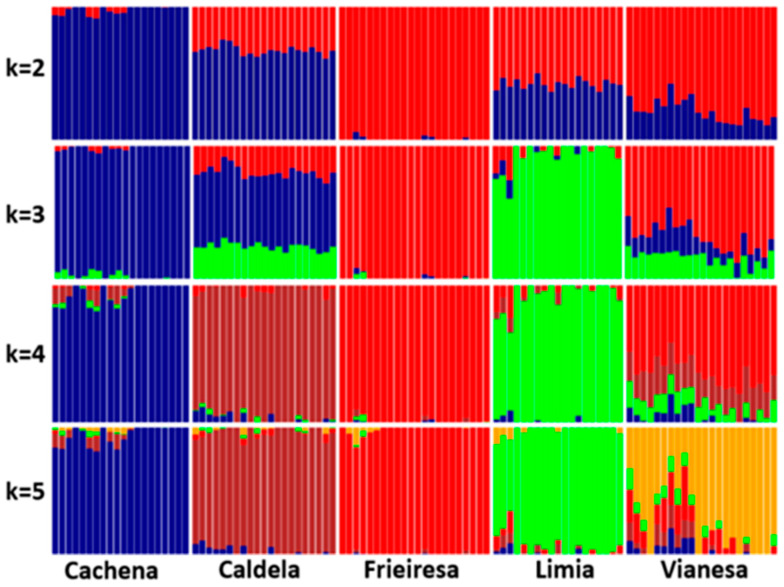
Population structure of Morenas Gallegas bovine breeds (Cachena, Caldelá, Frieiresa, Limiá, and Vianesa), inferred by using ADMIXTURE software. Each animal is represented by a single vertical bar divided into k colors, where k is the number of assumed ancestral clusters, which is graphically represented for k = 2–5.

The admixture plot with k ranging from 2 to 5 can be seen in Figure 3 and evidenced two differentiated clusters integrated by Cachena and Frieiresa appear in the first split (k = 2), while Caldelá, Limiá, and Vianesa showed different genetic influences from both clusters. At k = 3 and k = 4 Limiá and Caldelá formed separated groups while Vianesa evidenced a high level of admixture but with a higher influence of the group predominant in Frieiresa breed. At k = 5 Vianesa breed break into two clusters, one with evident admixture from Frieiresa and another cluster well differentiated from the other breeds.

**Figure 4:**
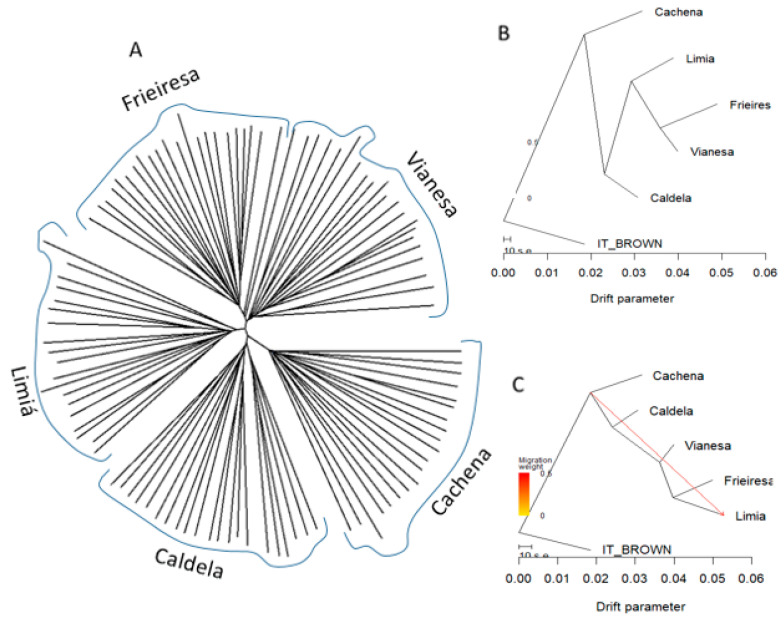
(**A**) Allele distance sharing dendrogram among Morenas Gallegas samples. (**B**) Maximum likelihood phylogenetic tree inferred using TreeMix with no migration rate and (**C**) with 1 migration edge allowed. Italian Brown Swiss (IT_BROWN) was used as outgroup in B and C trees.

Pairwise allele distance sharing (ASD) dendrogram showed a topography in agreement with admixture and PCA results (Figure 4A). The five breeds formed separated clusters although Frieiresa and Vianesa are closer among them than to the remaining breeds. The Neighbor-net graphs based on Reynolds genetic distance (Figure S3) showed a topography in agreement with admixture, PCA results, and ASD dendrogram. The five breeds formed separated clusters although Frieiresa and Vianesa are closer among them than to the remaining breeds.

In addition, TREEMIX phylogram without migration events showed a first separation event among Cachena and the remaining bovine breeds (Figure 4B). Subsequently, Caldelá formed an independent cluster and finally Limiá breed a third cluster while Vianesa and Frieiresa were very close to each other. When migration events were taken into account, the topology remains stable. The optimal number of migrations edges to add to the tree estimated with the SiZer method implemented in the optM function was 1, with a stronger basal signal connecting the origin of the tree with Limiá. (Figure 4C).

#### 3.2.2. Morenas Gallegas and Local and Cosmopolitan European Bovine Breeds

**Figure 5:**
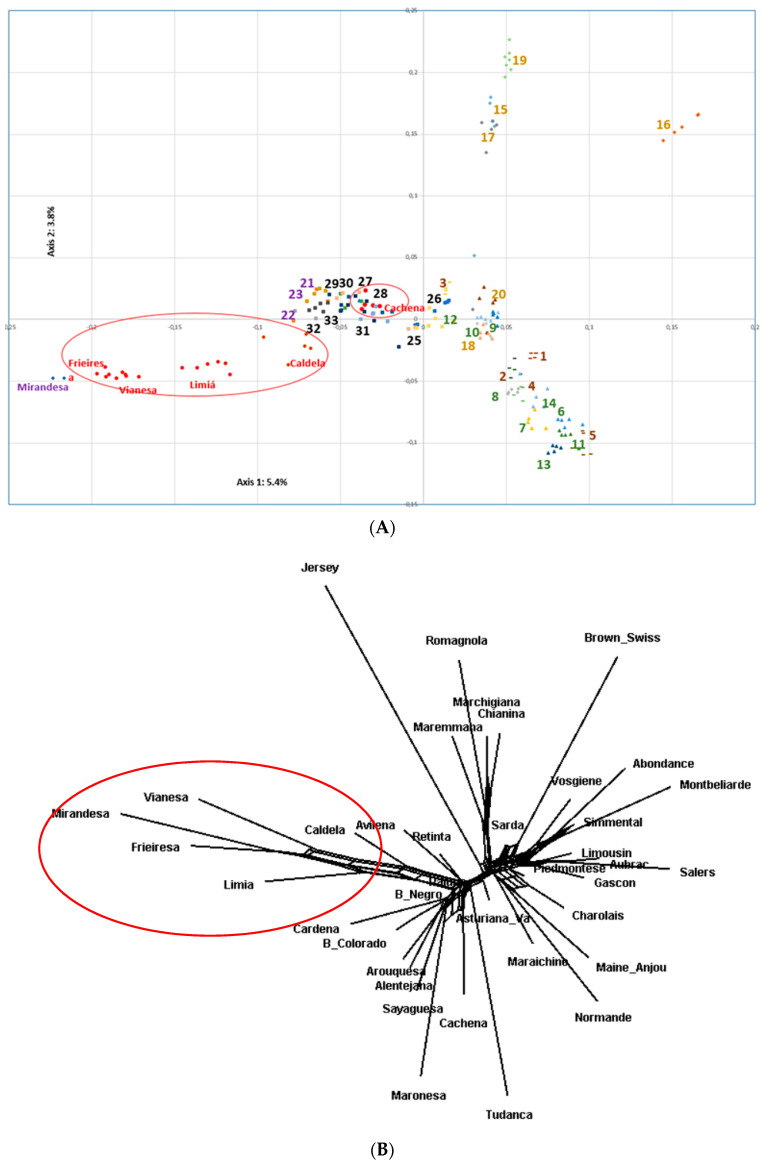
(**A**) PCA analysis among 38 European and Morenas Gallegas bovine breeds. Squares, triangles, and diamonds represent Iberian Peninsula (25: Asturiana de los Valles, 26: Avileña, 27: Berrenda en Colorado, 28: Berrenda en Negro, 29: Cardena, 30: Pajuna, 31: Retinta, 32: Sayaguesa, 33: Tudanca from Spain; 21: Alentejana, 22: Arouquesa, 23: Mirandesa, and 24: Maronesa from Portugal), French (6: Abondance, 7: Aubrac, 8: Gascon, 9: Maine Anjou, 10: Maraichine, 11: Montbeliarde, 12: Normande, 13: Salers, and 14: Vosgiene) and Italian bovine breeds (15: Chianina, 16: Marchigiana, 17: Maremmana, 18: Piedmontese, 19: Romagnola, and 20: Sarda), respectively. Lines reveal cosmopolitan ones (1: Brown Swiss, 2: Charolais, 3: Limousin, 4: Jersey, and 5: Simmental). Morenas Gallegas bovine breeds are represented by red circles and an external red line delimitate the Morenas Gallegas bovine breeds (34: Cachena, 35: Caldela, 36: Frieiresa, 37: Limiá, and 38: Vianesa). (**B**). Neighbor net dendrogram from Reynolds genetic distance from the same 38 European bovine breeds.

PCA and Neighbor-net dendogram of a set of local and cosmopolitan European bovine breeds analyzed jointly with Morenas Gaellgas can be found in Figure 5A,B. Three main clusters belonging to the geographic distribution appear in the Neighbor-net dendogram: Iberian Peninsula, Italy, and France. In addition, among Iberian Peninsula bovine breeds, Caldelá, Limiá, Vianesa, Frieiresa, and Mirandesa were grouped in separated clusters while Cachena was located closer to the remaining Iberian Peninsula bovine breeds.

**Figure 6:**
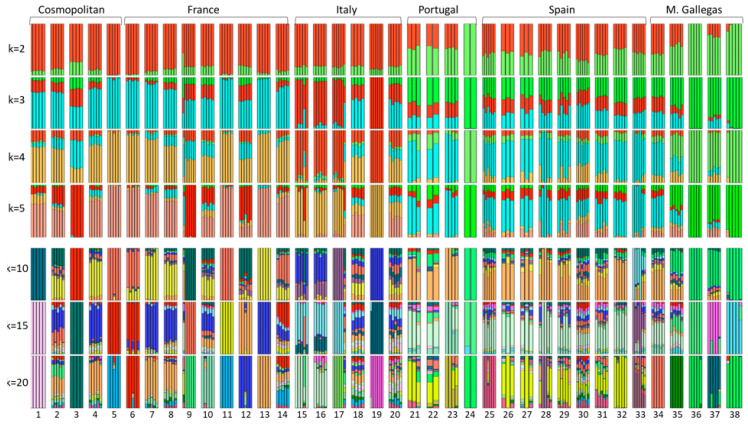
Admixture results among 38 European and the five Morenas Gallegas bovine breeds. Each animal is represented by a single vertical bar divided into k colors, where k is the number of assumed ancestral clusters, which is graphically represented for k = 2–5 and, 10, 15, 20. The equivalences of the breeds are the same as in Figure 5. Briefly: 24: Mirandesa; 34: Cachena; 35: Caldelá; 36: Frieiresa; 37: Limiá; 38: Vianesa.

ADMIXTURE results are shown in Figure 6. At lower k values (k = 2–5) values Frieiresa, Vianesa, and Mirandesa are grouped together, while Caldela evidenced an intermediate position between this group and the remaining Spanish, including Caldela, and Portuguese bovine breeds. The genetic relationships of Morenas Gallegas do not change significatively as k value increase.

The six migration events evidenced in the TREEMIX phylogram (Figure S4) showed a similar result to Reynolds genetic distance dendrogram (Figure 5B). One migration event was observed related to Morenas Gallegas from Maronesa to Cachena.

## 4. Discussion

The agricultural system in the Northwest of Spain during the 19th century favored fragmented multipurpose bovine populations (meat, milk, work, manure, etc.) adapted to local environmental conditions. Such bovine populations played an important role in familiar economies, but since the 20th century, the arrival of more productive bovine breeds and changes in the production systems led to the decline of those local breeds or even close to their extinction [4]. Consequently, at the end of the 20th century, Spanish government and local authorities launched conservation programs based on ex-situ-in vivo, ex situ-in vitro and in vitro actions to preserve these valuable genetic resources [7,20]. Although recent studies have showed positive evolution as a consequence of such conservation plans in the Morenas Gallegas bovine breeds [9], they are still at risk of extinction. The knowledge of the within breed genetic diversity and between breeds genetic relationships are important tools for enhancing efficient use of the breeds and for implementing conservation programs. The availability of high-throughput genotyping platforms has favored detailed analysis of cattle genetic diversity and SNPs have been used in a large number of articles focused on the analysis of genetic diversity within and among cattle breeds [21]. However, although the assessment of the genetic diversity of local breeds is an important task for conservation and management of local biodiversity and to avoid the loss of an important genetic resource, local breeds have been less analyzed than cosmopolitan ones. In addition, the adaptation of rare breeds to local environmental can represent a valuable source of genes [22]. Recently, several authors have successfully analyzed a set of local breeds in an European context [23,24].

In this study, the Morenas Gallegas bovine breeds were analyzed using genomic tools in order to elucidate their genetic diversity distribution and genetic relationships not only between them but also when comparing with other local and cosmopolitan European bovine breeds for the first time.

The results have evidenced the own genetic signatures of the Limiá, Frieiresa, Vianea, and Caldelá breeds, as revealed by the PCA and Reynolds genetic distance dendrograms which clearly separate them with respect to the Iberian Peninsula, Italian, and French bovine breeds analyzed that are grouped in different clusters in agreement with previous results [23,24]. The Morenas Gallegas bovine breeds are mainly located in mountainous rural areas that prevented their spread to other areas. Traditionally, Morenas Gallegas animals were bred in small herds of local farmers and for triple purpose (meat, milk, and work). The adaptation to local environments, the absence of genetic selection programmes, and the progressive replacement by commercial breeds more productive have favored their isolation from other bovine breeds. Furthermore, the reduced Morenas Gallegas breed sizes, could justify that the main genetic force that has influenced the genetic differentiation among the Morenas Gallegas bovine breeds is genetic drift. Cachena is the unique breed within the Morenas Gallegas group that clusters with the Spanish bovine breeds. This result can be justified due to the higher census and the lower inbreeding coefficients that Cachena breed shows among the Morenas Gallegas, so a lower genetic drift effect is expected to act. In addition, other bovine breed animals located in the same geographic region as the Cachena breed showed similar phenotypic characteristics in the first half of the 20th century, thus possible crosses among these may justify their lower genetic differentiation with other Spanish bovine breeds. Additionally, only one gene flow event was observed among the Morenas Gallegas breeds from Maronesa to Cachena that geographically are closely located.

Frieiresa and the Mirandesa Portuguese bovine breed share part of their geographical distribution area and it is well known their close genetic relationship (in fact both breeds are called with the same name in some geographic areas [25]), our results (PCA, ASD, and Reynolds genetic distance dendrograms) evidenced the close genetic proximity of both breeds. However, the analysis included two samples of Mirandesa breeds, so a higher sampling would be necessary to confirm the close genetic relationships between them.

Within-breed genetic diversity plays an important role in the short term for genetic improvement of productive traits and in the long term for conservation purposes. In 1990, local authorities launched a conservation plan of local breeds based on rotational mating systems, the development of a germplasm collection, and several demographic initiatives [8]. All the Morenas Gallegas bovine breeds analyzed show high or intermediate genetic diversity and Ne values. The Ne was for all the breeds higher than the threshold of 50 that can be considered at risk [26]. Furthermore, while until 13 generations ago, the Ne had a decreased tendency, recent studies based on genealogical information show in contrast positive Ne trends for all Morenas Gallegas breeds since 1996–2015 [9]. Therefore, our results evidenced that these conservation activities developed have been efficient in order to preserve the genetic diversity of Morenas Gallegas breeds. Some discrepancies were observed between genomic Ne and genealogical estimations; however, while most recent genomic values were from 13 generations ago, genealogical ones were current measures. In addition, genealogical information is highly dependent on the quality and depth of the genealogic data. Therefore, both facts could justify these discrepancies. Frieiresa breed showed the highest values of genomic inbreeding coefficients, (higher than 10%), based on the three ROH lengths and methods of moment. Thus, inbreeding monitoring is specifically significant in the Frieiresa breed which has the lowest census among the Morenas Gallegas bovine breeds.

Our results showed a high level of genetic differentiation among Morenas Gallegas bovine breeds. Caldelá and Cachena breeds evidenced the higher genetic differentiation level as revealed by ADMIXTURE, ASD, and Reynolds genetic distance dendrogram. Caldelá is the unique Morenas Gallegas breed belonging to the Black Iberian lineage origin, one of the three main historical bovine lineages in the Iberian Peninsula (Cantabrian, Iberian Black, and Chestnut) [21].

Furthermore, Cachena farmers traditionally selected animals with big horns, strong forehead, and reduced size (it is the smallest format of the Morenas Gallegas breed), well-adapted to work purposes in stony soils in which it is located, while the remaining Morenas Gallegas breeds did not have such definite selection procedures. Finally, Limiá, Vianesa, and Frieiresa breeds share the same ancestral lineage origin (Cantabrian) that may explain that they are genetically closer than to Cachena and Caldelá breeds, although preserving their genetic uniqueness.

It is remarkable the two genetic lineages evidenced within Vianesa animals (Figure 3). One lineage that showed an important ancestral Frieiresa component and a second one without it. The geographic proximity of both breeds could justify their genetic relationship. However, all the Morenas Gallegas breeds are located in a relatively small geographic region and the evident differentiation between the two lineages suggests other possible explanations that need further research.

## 5. Conclusions

The first analysis developed with genomic tools in the Morenas Gallegas, revealed the genetic uniqueness of the five breeds that encompass this racial group. Except Cachena breed, the remaining Morenas Gallegas are clearly separated from the remaining Spanish bovine breeds analyzed. Additionally, the five breeds have evidenced intermediate or high levels of genetic diversity, with Ne values, estimated from genomic tools, higher than 50 in all the breeds. However, the reduced census of the Frieiresa breed and its inbreeding coefficient (11%) suggests paying special attention in this breed. Finally, Vianesa breed shows two different lineages, one with an evident Frieiresa component and the other lacking it. Although the five breeds analyzed are included in a unique racial group, our results evidence clear genetic differences among them.

## Figures and Tables

**Table 1:** Breeds names and number of samples analyzed per breed. Effective population size (Ne), observed and expected heterozygosity (H_O_ and H_E_, respectively) and methods of moments inbreeding coefficient (F_HOM_).

Breed	Females	Males	N	Ne ^1^	F ^1^ (%)	Ne	Ho	He	F_HOM_
Cachena	3881	726	20	65	2.3	78	0.35	0.36	0.03
Caldelá	1288	176	21	74	3.1	63	0.36	0.36	0.02
Frieiresa	750	133	23	22	2.8	62	0.32	0.36	0.11
Limiá	1003	231	20	34	3.6	57	0.35	0.36	0.05
Vianesa	2303	422	22	32	3.6	69	0.34	0.36	0.06

^1^ Ne estimated from genealogical data adjusted to the genealogy information available and inbreeding coefficient based on genealogical information of the animals born since 2010. Data provided by the Federation of Galicia Local Breeds (BOAGA).

## Supplementary Materials

The following are available online at https://www.mdpi.com/2076-2615/10/11/1956/s1, Figure S1: Reynolds genetic distance dendrogram among the individuals of the five Morenas Gallegas bovine breeds, Figure S2: Inbreeding coefficients estimated from runs of homozygosity >4, >8, and >16 Mb, Figure S3: Reynolds genetic distance dendrogram among the individuals of the five Morenas Gallegas bovine breeds, Figure S4. Maximum likelihood phylogenetic tree inferred using TreeMix with 6 migration edge allowed, Table S1: Breeds, number of samples, geographic locations, and origin of the data of the bovine European samples included in the analysis. Table S2: Inbreeding coefficients estimated from plink (FHOM) and ROH > 4 Mb (F > 8 Mb) and >8 Mb (F > 8 Mb). Pearson correlation from FHOM and F > 4 Mb and F > 8 Mb.
