# Supplementary material for: Genomic Characterization of a Set of Iberian Peninsula Bovine Local Breeds at Risk of Extinction: Morenas Gallegas
Source: Animals (Basel). 2020 Oct 23;10(11):1956. doi: 10.3390/ani10111956 (PMC7690779; doi:10.3390/ani10111956)
Supplement: Supplementary file 1 [file animals-10-01956-s001.zip › animals-950901-supplementary.docx]

**Supplementary Material**

Supplementary Figure S1. Ne estimations from 13 to 98 generations ago for the five bovine breeds analysed: Cachena, Caldelá, Frieiresa, Limiá and Vianesa.


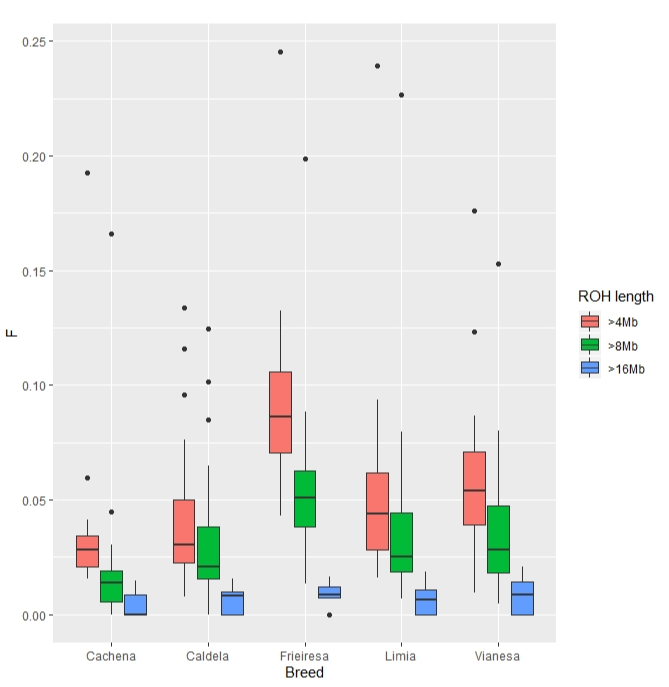


Supplementary Figure S2. Inbreeding coefficients estimated from runs of homozygosity >4, >8 and >16 Mb.


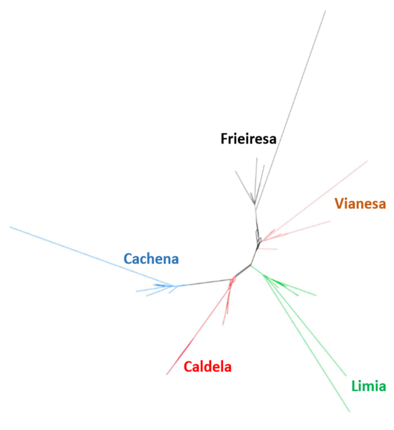


Supplementary Figure S3. Reynolds genetic distance dendrogram among the individuals of the five Morenas Gallegas bovine breeds, Cachena, Caldela, Frieiresa, Limiá and Vianesa.

Supplementary Figure S4


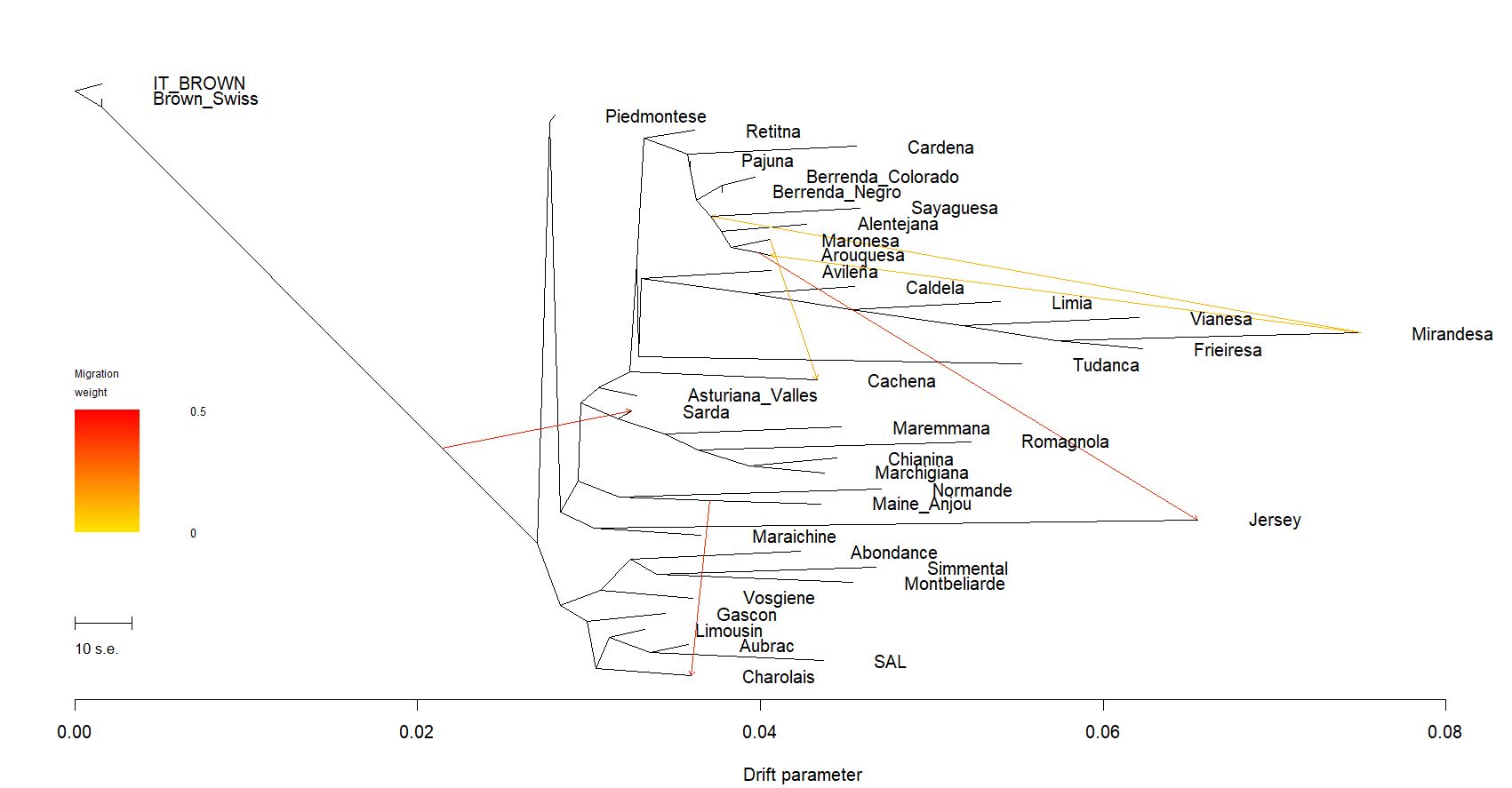


**Supplementary Figure S4**. Maximum likelihood phylogenetic tree inferred using TreeMix with 6 migration edge allowed.

Supplementary Table S1. Breeds, number of samples, geographic locations and origin of the data of the bovine European samples included in the analysis.

Upadhyay, M., Bortoluzzi, C., Barbato, M., Ajmone-Marsan, P., Colli, L., Ginja, C., Sonstegard, T. S., Bosse, M., Lenstra, J. A., Groenen, M., & Crooijmans, R. (2019). Deciphering the patterns of genetic admixture and diversity in southern European cattle using genome-wide SNPs. *Evolutionary applications*, *12*(5), 951–963. https://doi.org/10.1111/eva.12770

Mastrangelo, S., Ciani, E., Ajmone Marsan, P. *et al.* Conservation status and historical relatedness of Italian cattle breeds. *Genet Sel Evol* **50,**35 (2018). https://doi.org/10.1186/s12711-018-0406-x

Widde: http://widde.toulouse.inra.fr/widde/

| **Country** | | **ID** | **Breed** | **N** | **Data origin** |
| --- | --- | --- | --- | --- | --- |
| **Iberian Peninsula** | **Spain** | 25 | Asturiana de los Valles | 6 | This study |
|  |  | 26 | Avileña | 6 | This study |
|  |  | 27 | Berrenda_Colorado | 5 | Upadhyay et al, 2017 |
|  |  | 28 | Berrenda_Negro | 5 | Upadhyay et al, 2017 |
|  |  | 34 | Cachena | 5 | This study |
|  |  | 35 | Caldelá | 5 | This study |
|  |  | 29 | Cardena | 5 | Upadhyay et al, 2017 |
|  |  | 36 | Frieiresa | 5 | This study |
|  |  | 37 | Limiá | 5 | This study |
|  |  | 30 | Pajuna | 5 | Upadhyay et al, 2017 |
|  |  | 31 | Retinta | 5 | This study |
|  |  | 32 | Sayaguesa | 5 | Upadhyay et al, 2017 |
|  |  | 33 | Tudanca | 6 | This study |
|  |  | 38 | Vianesa | 5 | This study |
|  | **Portugal** | 21 | Alentejana | 2 | Upadhyay et al, 2017 |
|  |  | 22 | Arouquesa | 3 | Upadhyay et al, 2017 |
|  |  | 23 | Maronesa | 4 | Upadhyay et al, 2017 |
|  |  | 24 | Mirandesa | 2 | Upadhyay et al, 2017 |
| **Italy** | | 15 | Chianina | 5 | Mastrangelo et al., 2018 |
|  |  | 16 | Marchigiana | 5 | Mastrangelo et al., 2018 |
|  |  | 17 | Maremmana | 5 | Mastrangelo et al., 2018 |
|  |  | 18 | Piedmontese | 5 | Mastrangelo et al., 2018 |
|  |  | 19 | Romagnola | 5 | Mastrangelo et al., 2018 |
|  |  | 20 | Sarda | 5 | Mastrangelo et al., 2018 |
| **France** | | 6 | Abondance | 5 | Widde |
|  |  | 7 | Aubrac | 5 | Widde |
|  |  | 8 | Gascon | 5 | Widde |
|  |  | 9 | Maine Anjou | 5 | Widde |
|  |  | 11 | Montbeliarde | 5 | Widde |
|  |  | 12 | Normande | 5 | Widde |
|  |  | 13 | Salers | 5 | Widde |
|  |  | 14 | Vosgienne | 5 | Widde |
|  |  | 10 | Maraichine | 5 | Widde |
| **Cosmopolitan** | | 1 | Brown Swiss | 5 | Widde |
|  |  | 2 | Charolais | 5 | Widde |
|  |  | 3 | Jersey | 5 | Upadhyay et al, 2017 |
|  |  | 4 | Limousin | 5 | Mastrangelo et al., 2018 |
|  |  | 5 | Simmental | 5 | Widde |

Supplementary Table S2

.

| **Breed** | **F_HOM_** | **F_>4Mb_** | **F_>8Mb_** |
| --- | --- | --- | --- |
| Cachena | 0,03 | 0,04 | 0,02 |
| Caldela | 0,02 | 0,04 | 0,03 |
| Frieiresa | 0,11 | 0,09 | 0,06 |
| Limia | 0,05 | 0,06 | 0,04 |
| Vianesa | 0,06 | 0,06 | 0,04 |
| Pearson Correlation |  | 0,99 | 0,89 |

**Supplementary Table S2.** Inbreeding coefficients estimated from plink (F_HOM_) and ROH >4Mb (F_>8Mb_) and >8Mb (F_>8Mb_). Pearson correlation from F_HOM_ and F_>4Mb_ and F_>8Mb_.
